# Improved Glycaemic Control with Biphasic Insulin Aspart 30 in Type 2 Diabetes Patients Failing Oral Antidiabetic Drugs: PRESENT Study Results

**DOI:** 10.1111/j.1753-5174.2008.00015.x

**Published:** 2009-06

**Authors:** Serdar Güler, Surendra Kumar Sharma, Majeed Almustafa, Chong Hwa Kim, Sami Azar, Rucsandra Danciulescu, Marina Shestakova, Duma Khutsoane, Ole Molskov Bech

**Affiliations:** * Ankara Numune Training and Research HospitalAnkara, Turkey; † M G Medical CollegeJaipur, India; ‡ Al Mstansiriya UniversityBaghdad, Iraq; § Sejong General HospitalBucheon City, South Korea; ¶ American University of BeirutBeirut, Lebanon; ** N. Paulescu InstituteBucharest, Romania; †† Federal Scientific Centre of EndocrinologyMoscow, Russia; ‡‡ Medi-ClinicBloemfontein, South Africa; §§ Novo Nordisk International Operations Clinical Development CentreBeijing, China

**Keywords:** Biphasic Insulin Aspart 30, Oral Antidiabetic Drug Failure, Type 2 Diabetes

## Abstract

**Aims:**

This paper presents the treatment outcomes for patients intiated on biphasic insulin aspart 30 (BIAsp 30) treatment: BIAsp 30-only, BIAsp 30 + sulphonylureas (SU), BIAsp 30 + biguanides (BI), BIAsp 30 + SU + BI, BIAsp 30 + alpha-glucosidase inhibitors (GI), and BIAsp 30 + BI + thiazolidinediones (TZD) after failing oral antidiabetic drugs (OADs) treatment.

**Methods:**

This was a multi-national, multi-centre, six-month, prospective, open-labelled, uncontrolled, clinical experience evaluation study, with the exception of a three-month study in one country (China) (“all exclude China” and “China”). Initiation and discontinuation of BIAsp 30 treatment were entirely at the discretion of the attending physicians.

**Results:**

Mean HbA_1c_, FPG and PPPG were significantly reduced from baseline at three and six months in all groups (*P* < 0.001). In “all exclude China”, reductions in mean HbA_1c_, FPG and PPPG at six months were as follows: BIAsp 30-only group (−2.12 ± 1.76% points; −4.82 ± 3.86 mmol/L; −6.89 ± 4.74 mmol/L), BIAsp 30 + BI group (−2.24 ± 1.77% points; −4.48 ± 3.68 mmol/L; −6.66 ± 4.55 mmol/L), BIAsp 30 + SU group (−1.95 ± 1.59% points; −3.98 ± 3.19 mmol/L; −6.25 ± 4.45 mmol/L) and BIAsp 30 + SU + BI group (−1.78 ± 1.20% points; −3.57 ± 2.78 mmol/L; −5.89 ± 3.98 mmol/L). The only serious adverse drug reaction was reported by the BIAsp 30-only group. In the “China” group, reductions in mean HbA_1c_, FPG and PPPG at three months were: BIAsp 30-only group (−2.16 ± 1.52% points; −3.34 ± 2.49 mmol/L; −6.29 ± 3.92 mmol/L), BIAsp 30 + BI group (−2.44 ± 1.52% points; −4.01 ± 2.50 mmol/L; −7.10 ± 3.96 mmol/L), BIAsp 30 + GI group (−2.33 ± 1.41% points; −4.34 ± 2.52 mmol/L; −7.97 ± 3.99 mmol/L) and BIAsp 30 + BI + TZD group (−1.21 ± 1.60% points; −3.50 ± 2.29 mmol/L; −5.97 ± 3.39 mmol/L). No serious ADR were reported in China. The most frequent hypoglycaemic episodes were diurnal and minor in nature.

**Conclusions:**

BIAsp 30 treatment in a clinical setting improved glycaemic control in type 2 diabetes patients failing OADs.

## Introduction

The progressive nature of type 2 diabetes mellitus will require most patients to intensify their therapies in order to maintain good glycaemic control in the longer term [2]. Intensification of therapy has been shown to reduce the risk of many complications associated with type 2 diabetes [3]. There are various options for initiating insulin treatment in insulin-naïve patients. According to the recent consensus statement from the American Diabetes Association (ADA) and the European Association for the Study of Diabetes (EASD), patients experiencing poor glycaemic control with lifestyle interventions and oral antidiabetic drugs (OADs) should intensify their treatment with the addition of insulin [4]. For newly diagnosed patients with severely uncontrolled diabetes with catabolism, fasting plasma glucose (FPG) levels above 13.9 mmol/L, random glucose levels consistently above 16.7 mmol/L or HbA_1c_ above 10%, insulin therapy in combination with lifestyle change is the treatment of choice [4].

Insulin is effective in lowering hyperglycaemia, with potential beneficial effects on triglyceride and HDL cholesterol levels [4,5]. Its disadvantage remains its potential to induce weight gain and cause hypoglycaemia. Patients treated with a combination of insulin + OADs have benefited from the insulin-sparing effects of some OADs, which allow a lower dose of insulin to be used, thereby reducing the potential for hypoglycaemia [2,6]. A Cochrane review of 20 randomised controlled trials has shown that insulin + OAD combination therapy was associated with a 43% relative reduction in total daily insulin requirement, compared with insulin monotherapy [7]. The choice of glycaemic goals and the medications used to achieve them must be individualised for each patient, balancing the potential for lowering HbA_1c_ and anticipated long-term benefit with specific safety issues and other characteristics of regimens, including side effects, tolerability, patient burden, long-term adherence, cost and the effects of the medications on hypertension and dyslipidaemia [4].

Biphasic insulin aspart 30 (BIAsp 30; NovoMix® 30, Novo Nordisk, Bagsvaerd, Denmark) is a premixed insulin analogue that contains both rapid-acting insulin aspart and long-acting protaminated insulin aspart in the ratio 30:70 respectively. Its safety and efficacy have been demonstrated in numerous randomised clinical trials [8–15]. It has been used successfully alone or in combination with the majority of marketed OADs, such as biguanides (BI) [8,16] and thiazolidinediones (TZD) [17].

While the efficacy and safety of BIAsp 30 in randomised controlled clinical trials have been established in the literature, data on its use in routine clinical practice situations remain limited. The Physicians' Routine Evaluation of Safety and Efficacy of NovoMix® 30 Therapy (PRESENT) Study is the largest, multi-national, observational study on BIAsp 30 in real clinical settings completed to date. It aims to collect data complementary to the published clinical data on the safety and efficacy of BIAsp 30 use in type 2 diabetes. In this article, we present results from a subgroup poorly controlled (with HbA_1c_ ≥ 7% at baseline) on OADs and who either added BIAsp 30 treatment to their existing OADs or transferred to BIAsp 30 monotherapy. The patient data were categorised according to the type of therapy received throughout the study, i.e., BIAsp 30 only or combination of BIAsp 30 with OADs (sulphonylureas or biguanides or sulphonylureas+biguanides).

## Patients and Methods

### Study Design and Treatment

This was a multi-national, multi-centre, six-month, prospective, open-labelled, uncontrolled, clinical experience evaluation study. The objective of this observational study was to evaluate the efficacy and safety of using BIAsp 30, as a monotherapy or in combination with OADs, for type 2 diabetes management in routine clinical practice. The requirement for ethics committee approval or patient informed consent for this study was according to local regulations. In two participating countries, ethics committee approval was required but patient informed consent was not required. In another two participating countries, both ethics committee approval and patient informed consent were required. For the other countries, both ethics committee and patient informed consent approvals were not obtained as these were not required. As this was an observational study, the initiation and discontinuation of BIAsp 30 treatment were entirely at the discretion of the attending physicians and no intervention was added to the physician's routine practice. Except for patients in one country, all patients purchased their own biphasic insulin aspart 30 as in routine clinical practice. No special investigational procedures outside clinical practice were planned.

### Patient Inclusion and Exclusion Criteria

As this was an observational study, the only criteria were that patients had type 2 diabetes mellitus, were inadequately controlled on their current therapy and were prescribed BIAsp 30 as monotherapy or in combination with OADs in accordance with the approved labelling. In this paper, we present data from insulin-naïve patients with a baseline HbA_1c_ of ≥7% who were previously treated only with OADs.

### Participating Countries

This study was planned for 15 countries [18]: China (N = 11,724), India (N = 3,560), Iraq (N = 2,031), Jordan (N = 380), Lebanon (N = 685), Romania (N = 1,227), Russia (N = 2,256), Saudi Arabia and the Gulf countries (N = 2,228) which includes Kuwait, Qatar and the United Arab Emirates, South Africa (N = 1,510), South Korea (N = 831), Sri Lanka (N = 81), and Turkey (N = 3,149). However, data from Sri Lanka were excluded from this paper because data were collected only at baseline. The data presented here are from the remaining 14 countries, with data from China presented separately as data were collected for 3 months only.

### Data Collection and Study Endpoints

The efficacy endpoints were the changes in HbA_1c_, FPG and postprandial plasma glucose (PPPG) at the end of treatment from baseline. The safety endpoints were the occurrence of hypoglycaemic episodes and adverse drug reactions (ADRs). Patient data were collected at baseline, three months and six months using standardised forms. These were patient demography (at baseline), weight, duration of diabetes, current diabetes therapy, HbA_1c_, FPG and PPPG measurements, number of hypoglycaemic episodes and ADRs. Hypoglycaemic episodes and ADRs were based on patient recollection and clinical records from three months prior to baseline visit and from the last visit for three- and six-month datapoints. Major episodes were defined as those where the patient was unable to perform self-treatment, i.e., glucose had to be administered to the patient by another person. Daytime (diurnal) episodes occurred between 06:00 to 00:00.

### Statistical Analyses

The patients were categorised into groups according to the therapies received constantly during the study: BIAsp 30-only, BIAsp 30 + sulphonylureas (SU), BIAsp 30 + biguanides (BI), BIAsp 30 + SU + BI, BIAsp 30 + alpha glucosidase inhibitors (GI), and BIAsp 30 + BI + thiazolidinediones (TZD). These BIAsp 30 + OAD combinations had the largest sample sizes in the study population. Patients who had changes in therapy during the study period were not included in this sub-analysis. Other combinations were omitted because their sample sizes were too small for meaningful analysis. The safety analysis set consisted of enrolled patients with baseline data. Hence, patients who withdrew from the study but who have baseline data are included in the analysis and contribute with whatever data that is available. Baseline demographic information, diabetes therapy, and efficacy and safety outcomes were presented as descriptive statistics (%, mean ± S.D. and 95% confidence interval). Changes in HbA_1c_, FPG and PPPG from baseline were analysed using the paired t-test. Baseline and demographic variables (patients' country, gender, weight, body mass index [BMI], ethnicity, age, duration of diabetes, HbA_1c_ at baseline, total daily dose of BIAsp 30 per body weight [bw], previous OAD treatment) and current treatment were fitted into a generalised linear model to determine the factors that affected the change in HbA_1c_ at six months from baseline. Hypoglycaemic episodes and ADRs were presented according to category and severity using summary statistics and event rates. All the statistical analyses were performed using SAS® version 9.1.3 (SAS Institute, NC, USA).

## Results

### Subject Disposition, Baseline Demography and Prior OAD Exposure

Table 1 shows the disposition of subjects by the type of therapy during the study. The safety population in the BIAsp 30-only group (N = 2,507 and 2,464) was the largest compared with the other BIAsp 30 + OAD combination in both the “all exclude China” and “China” groups (Table 1). In “all exclude China”, the majority of patients completed the six-month study (88% to 91% across the groups), while a small proportion continued the study until three months (9% to 13% across the groups). Baseline BMI, HbA_1c_, FPG and PPPG were lowest in the BIAsp 30 + SU + BI group (Table 2a). The most common prior OAD treatments among the groups were SU + BI and SU-only (Table 3a). In the “China” group, baseline BMI, HbA_1c_, and FPG were lowest in the BIAsp 30-only group while PPPG was low in both the BIAsp 30-only and BIAsp 30 + BI + TZD groups (Table 2b). The most common prior OAD treatments among the groups were SU + BI and BI-only (Table 3b). Since the dosage of SU was not analysed due to the many types available, the dosage of OADs used during the study will not be discussed.

**Table 3:** (a)OAD therapy prior to the study—all exclude China; (b)OAD therapy prior to the study—China

	BIAsp 30 only	BIAsp 30 + SU	BIAsp 30 + BI	BIAsp 30 + SU + BI
	
	N (% patients)			
Safety population	2,507	269	1,062	489
SU	670 (26.7)	157 (58.4)	73 (6.9)	21 (4.3)
BI	100 (4.0)	1 (0.4)	126 (11.9)	4 (0.8)
GI	18 (0.7)	0 (0.0)	1 (0.1)	1 (0.2)
TZD	11 (0.4)	1 (0.4)	4 (0.4)	0 (0.0)
MEG	28 (1.1)	3 (1.1)	2 (0.2)	0 (0.0)
Combinations of OADs				
SU + BI	959 (38.3)	73 (27.1)	566 (53.3)	400 (81.8)
SU + BI + TZD	123 (4.9)	6 (2.2)	82 (7.7)	28 (5.7)
Other combinations	598 (23.9)	28 (10.4)	208 (19.5)	35 (7.2)

	BIAsp 30 only	BIAsp 30 + BI	BIAsp 30 + GI	BIAsp 30 + BI + TZD
	
	N (% patients)			

Safety population	2,464	541	165	156
SU	464 (18.8)	47 (8.7)	16 (9.7)	4 (2.6)
BI	433 (17.6)	162 (29.9)	9 (5.5)	5 (3.2)
Alpha-GI	167 (6.8)	1 (0.2)	27 (16.4)	1 (0.6)
TZD	59 (2.4)	4 (0.7)	0	0
MEG	203 (8.2)	4 (0.7)	3 (1.8)	0
Combinations of OADs				
SU + BI	524 (21.3)	217 (40.1)	33 (20.0)	14 (9.0)
SU + BI + TZD	14 (0.6)	4 (0.7)	2 (1.2)	101 (64.7)
Other combinations	600 (24.4)	102 (18.9)	75 (45.5)	31 (19.9)

BI = biguanides; GI = glucosidase inhibitors; MEG = meglitinides; SU = sulphonylureas; TZD = thiazolidinediones.

**Table 2:** (a)Baseline characteristics—all exclude China; (b)Baseline characteristics—China

Characteristics	BIAsp 30 only	BIAsp 30 + SU	BIAsp 30 + BI	BIAsp 30 + SU + BI
Safety population	2,507	269	1,062	489
Gender, N (% males)	2,492 (49.7)	267 (40.8)	1,057 (48.0)	487 (59.8)
Ethnicity, N	2,474	266	1,023	483
Asian,* %	38.4	54.1	31.4	84.9
White, %	36.0	34.2	32.6	5.0
Middle Eastern,† %	21.0	10.5	25.8	8.7
Other, %	4.6	1.2	10.2	1.4
Mean age, N; years ± SD	2,056; 55.8 ± 11.6	224; 56.2 ± 11.2	845; 53.8 ± 11.3	410; 55.9 ± 10.6
Mean diabetes duration, N; years ± SD	2,373; 9.0 ± 6.1	244; 8.7 ± 6.6	989; 8.5 ± 5.9	467; 9.2 ± 5.7
Mean BMI,N; kg/m^2^ ± SD	2,448; 26.6 ± 4.7	266; 26.5 ± 4.9	1,032; 29.1 ± 5.2	485; 26.0 ± 4.5
Mean HbA_1c_, N; % ± SD	2,507; 9.8 ± 1.8	269; 9.5 ± 1.6	1,062; 9.9 ± 1.8	489; 9.3 ± 1.5
Mean FPG, N; Mmol/L ± SD	2,428; 13.0 ± 4.1	261; 11.8 ± 3.4	1,018; 12.4 ± 3.7	488; 10.7 ± 3.0
Mean PPPG, N; mmol/L ± SD	2,392; 17.6 ± 4.8	255; 16.8 ± 4.3	1,008; 16.9 ± 4.5	483; 15.7 ± 4.0

Characteristics	BIAsp 30 only	BIAsp 30 + BI	BIAsp 30 + GI	BIAsp 30 + BI + TZD

Safety population	2,464	541	165	156
Gender, N (% males)	1,396 (56.7)	301 (55.6)	78 (47.3)	81 (51.9)
Ethnicity, N	2,462	540	165	156
Asian,* %	100	99.8	100	100
White, %	<0.1%	0.2	0	0
Mean age, N; years ± SD	2,448; 54.1 ± 10.9	533; 53.9 ± 11.0	164; 57.6 ± 10.6	154; 58.0 ± 10.8
Mean diabetes duration, N; years ± SD	2,424; 5.2 ± 4.2	529; 5.6 ± 3.8	162; 5.5 ± 4.1	153; 5.6 ± 3.4
Mean BMI,N; kg/m^2^ ± SD	2,461; 23.9 ± 2.7	537; 24.6 ± 2.7	164; 24.2 ± 2.7	156; 25.1 ± 1.9
Mean HbA_1c_, N; % ± SD	2,464; 9.1 ± 1.6	541; 9.6 ± 1.9	165; 9.2 ± 1.6	156; 9.2 ± 1.4
Mean FPG, N; mmol/L ± SD	2,462; 10.5 ± 2.6	541; 11.4 ± 2.9	165; 11.5 ± 2.6	145; 10.8 ± 2.4
Mean PPPG, N; mmol/L ± SD	2,463; 15.4 ± 4.0	541; 16.6 ± 4.2	165; 17.4 ± 4.0	155; 15.3 ± 3.8

* Asian includes Pacific Islander.

† Middle Eastern includes Arab.

BI = biguanides; SU = sulphonylureas; FPG = fasting plasma glucose; PPPG = postprandial plasma glucose; GI = alpha-glucosidase inhibitors; TZD = thiazolidinediones.

**Table 1:** Subject disposition

	All exclude China	China
Enrolled	22,857	11,724
Safety population	21,977	11,662
OAD therapy only and HbA_1c_ ≥ 7%	8,151	4,551
Therapy during study*		
BIAsp 30 only	2,507	2,464
BIAsp 30 + BI	1,062	541
BIAsp 30 + SU	269	NA
BIAsp 30 + SU + BI	489	NA
BIAsp 30 + GI	NA	165
BIAsp 30 + BI + TZD	NA	156

* Only presented for the four most common type of therapy.

BI = biguanides; GI = glucosidase inhibitors; MEG = meglitinides; NA = not applicable; SU = sulphonylureas; TZD = thiazolidinediones.

### BIAsp 30 Exposure During Study

BIAsp 30 dosage per body weight at three and six months increased from baseline in all the groups (Table 4a, b). At all visits in the “all exclude China” group, the mean dosage was higher in the BIAsp 30-only and BIAsp 30 + BI groups and lower in the BIAsp 30 + SU and BIAsp 30 + SU + BI groups. In all the groups, the majority of patients followed a twice-daily injection regimen of BIAsp 30. However, a substantial proportion of patients in the BIAsp 30 + SU and BIAsp 30 + SU + BI groups injected only once daily. In the “China” group, at all visits, the mean dosage was highest in the BIAsp 30 + BI + TZD group (Table 4b). In all the groups, the majority of patients followed a twice-daily injection regimen of BIAsp 30.

**Table 4:** (a)Daily BIAsp 30 dosage and number of injections at baseline, three months and six months—all exclude China; (b)Daily BIAsp 30 dosage and number of injections at baseline and three months—China

	BIAsp 30 only	BIAsp 30 + SU	BIAsp 30 + BI	BIAsp 30 + SU + BI
Safety population	2,507	269	1,062	489
At treatment initiation				
Mean total dose, N; U/kg bw ± S.D.	2,465	264	1,044	485
	0.47 ± 0.19	0.29 ± 0.15	0.42 ± 0.18	0.30 ± 0.15
Once daily, N (%) patients	277 (11.0)	126 (47.4)	165 (15.7)	218 (44.6)
Twice daily, N (%) patients	2,175 (86.9)	138 (51.9)	871 (82.5)	270 (55.3)
Thrice daily, N (%) patients	50 (2.0)	2 (0.8)	20 (1.9)	0 (0.0)
At three months				
Mean total dose, N; U/kg bw ± S.D.	2,283	254	934	464
	0.53 ± 0.21	0.34 ± 0.17	0.48 ± 0.18	0.34 ± 0.16
Once daily, N (%) patients	240 (9.9)	112 (43.1)	113 (11.4)	182 (38.1)
Twice daily, N (%) patients	2,102 (86.9)	145 (55.8)	836 (84.8)	290 (60.6)
Thrice daily, N (%) patients	76 (3.1)	3 (1.2)	37 (3.8)	6 (1.3)
At six months				
Mean total dose, N; U/kg bw ± S.D.	2,129	235	910	401
	0.55 ± 0.21	0.38 ± 0.19	0.53 ± 0.20	0.36 ± 0.18
Once daily, N (%) patients	220 (9.9)	91 (37.9)	97 (10.1)	161 (37.7)
Twice daily, N (%) patients	1,860 (84.0)	142 (59.2)	805 (83.9)	259 (60.8)
Thrice daily, N (%) patients	134 (6.1)	7 (2.9)	58 (6.0)	6 (1.4)

	BIAsp 30 only	BIAsp 30 + BI	BIAsp 30 + GI	BIAsp 30 + BI + TZD

Safety population	2,464	541	165	156
At treatment initiation				
Mean total dose, N; U/kg bw ± S.D.	2,462	539	164	156
	0.44 ± 0.15	0.42 ± 0.14	0.43 ± 0.16	0.49 ± 0.10
Once daily, N (%) patients	0	0	0	0
Twice daily, N (%) patients	2,371 (96.2)	498 (92.1)	160 (97.0)	145 (92.9)
Thrice daily, N (%) patients	93 (3.8)	43 (7.9)	5 (3.0)	11 (7.1)
At three months				
Mean total dose, N; U/kg bw ± S.D.	2,462	541	164	156
	0.48 ± 0.15	0.46 ± 0.14	0.48 ± 0.14	0.58 ± 0.15
Once daily, N (%) patients	0	0	0	0
Twice daily, N (%) patients	2,323 (94.3)	497 (91.9)	161 (97.6)	151 (96.8)
Thrice daily, N (%) patients	141 (5.7)	44 (8.1)	4 (2.4)	3 (1.9)

BI = biguanides; SU = sulphonylureas; GI = alpha-glucosidase inhibitors; TZD = thiazolidinediones.

Percentages may not total 100% because of rounding off.

### Efficacy

In the “all exclude China” group, mean HbA_1c_, FPG and PPPG were significantly reduced from baseline at three and six months in all groups (*P* < 0.001) (Table 5a). Reductions in mean HbA_1c_, FPG and PPPG after 6 months were as follows: BIAsp 30-only (−2.12 ± 1.76% points, −4.82 ± 3.86 mmol/L and −6.89 ± 4.74 mmol/L), BIAsp 30 + BI group (−2.24 ± 1.77% points, −4.48 ± 3.68 mmol/L and −6.66 ± 4.55 mmol/L), BIAsp 30 + SU group (−1.95 ± 1.59% points, −3.98 ± 3.19 mmol/L and −6.25 ± 4.45 mmol/L) and BIAsp 30 + SU + BI group (−1.78 ± 1.20% points, −3.57 ± 2.78 mmol/L and −5.89 ± 3.98 mmol/L). The proportion of patients who achieved an HbA_1c_ of less than 7% at six months was as follows: BIAsp 30 + SU (29%), BIAsp 30 + BI (29%), BIAsp 30 + SU + BI groups (31%), and BIAsp 30-only group (23%). Further, a small proportion of patients achieved target HbA_1c_ without reporting hypoglyceamic episodes (ranging from 21% to 32% across the groups). Based on the generalised linear model, patients' country, ethnicity, age, baseline HbA_1c_ and total daily dose of BIAsp 30 per bw were found to have significant effects on the change in HbA_1c_ at six months from baseline (*P* < 0.01).

**Table 5:** (a)Change in glucose parameters from baseline—all exclude China; (b)Change in glucose parameters from baseline—China

	BIAsp 30 only	BIAsp 30 + SU	BIAsp 30 + BI	BIAsp 30 + SU + BI
Safety population	2,507	269	1,062	489
Mean HbA_1c_, N; % ± SD (95% CI)				
At baseline	2,507	269	1,062	489
	9.84 ± 1.78	9.53 ± 1.60	9.91 ± 1.79	9.34 ± 1.45
Change at three months of treatment	1,778	182	674	339
	−1.58 ± 1.61*	−1.76 ± 1.45*	−1.91 ± 1.77*	−1.23 ± 1.07*
	(−1.66; −1.51)	(−1.97; −1.55)	(−2.04; −1.77)	(−1.35; −1.12)
Change at six months of treatment	2,169	238	929	425
	−2.12 ± 1.76*	−1.95 ± 1.59*	−2.24 ± 1.77*	−1.78 ± 1.20*
	(−2.19; −2.04)	(−2.16; −1.75)	(−2.35; −2.12)	(−1.89; −1.66)
Mean FPG, N; mmol/L ± SD (95% CI)				
At baseline	2,428	261	1,018	488
	13.00 ± 4.06	11.75 ± 3.37	12.42 ± 3.67	10.73 ± 3.00
Change at three months of treatment	2,262	250	935	478
	−4.05 ± 3.82*	−3.22 ± 2.92*	−3.80 ± 3.65*	−2.78 ± 2.48*
	(−4.20; −3.89)	(−3.58; −2.85)	(−4.04; −3.57)	(−3.01; −2.56)
Change at six months of treatment	2,088	233	896	427
	−4.82 ± 3.86*	−3.98 ± 3.19*	−4.48 ± 3.68*	−3.57 ± 2.78*
	(−4.99; −4.66)	(−4.39; −3.57)	(−4.72; −4.24)	(−3.83; −3.31)
Mean PPPG, N; mmol/L ± SD (95% CI)				
At baseline	2,392	255	1,008	483
	17.56 ± 4.82	16.75 ± 4.34	16.93 ± 4.46	15.71 ± 4.02
Change at three months of treatment	2,231	230	919	472
	−5.87 ± 4.88*	−5.32 ± 4.12*	−5.73 ± 4.61*	−4.42 ± 3.55*
	(−6.07; −5.67)	(−5.85; −4.78)	(−6.03; −5.43)	(−4.74; −4.10)
Change at six months of treatment	2,042	229	888	421
	−6.89 ± 4.74*	−6.25 ± 4.45*	−6.66 ± 4.55*	−5.89 ± 3.98*
	(−7.09; −6.68)	(−6.83; −5.67)	(−6.96; −6.36)	(−6.27; −5.51)

	BIAsp 30 only	BIAsp 30 + BI	BIAsp 30 + GI	BIAsp 30 + BI + TZD

Safety population	2,464	541	165	156
Mean HbA_1c_, N; % ± SD (95% CI)				
At baseline	2,464	541	165	156
	9.08 ± 1.63	9.62 ± 1.93	9.21 ± 1.58	9.24 ± 1.40
Change at three months of treatment	2,463	541	165	156
	−2.16 ± 1.52*	−2.44 ± 1.52*	−2.33 ± 1.41*	−1.21 ± 1.60*
	(−2.22; −2.10)	(−2.57; −2.31)	(−2.55; −2.11)	(−1.47; −0.96)
Mean FPG, N; mmol/L ± SD (95% CI)				
At baseline	2,462	541	165	145
	10.47 ± 2.61	11.39 ± 2.91	11.53 ± 2.63	10.75 ± 2.42
Change at three months of treatment	2,454	541	165	145
	−3.34 ± 2.49*	−4.01 ± 2.50*	−4.34 ± 2.52*	−3.50 ± 2.29*
	(−3.43; −3.24)	(−4.22; −3.80)	(−4.73; −3.95)	(−3.87; −3.12)
Mean PPPG, N; mmol/L ± SD (95% CI)				
At baseline	2,463	541	165	155
	15.44 ± 4.03	16.60 ± 4.21	17.43 ± 4.04	15.28 ± 3.81
Change at three months of treatment	2,459	541	165	155
	−6.29 ± 3.92*	−7.10 ± 3.96*	−7.97 ± 3.99*	−5.97 ± 3.39*
	(−6.44; −6.13)	(−7.44; −6.77)	(−8.58; −7.35)	(−6.51; −5.43)

* *P* < 0.001 (change from baseline; paired t-test).

BI = biguanides; CI = confidence interval; FPG = fasting plasma glucose; PPPG = postprandial plasma glucose SU = sulphonylureas; GI = alpha-glucosidase inhibitors; TZD = thiazolidinediones.

To convert mmol/L to mg/dL, multiply by 18.

In the “China” group, mean HbA_1c_, FPG and PPPG were significantly reduced from baseline at three months in all groups (*P* < 0.001) (Table 5b). Reductions in mean HbA_1c_, FPG and PPPG after 3 months were as follows: BIAsp 30-only (−2.16 ± 1.52% points, −3.34 ± 2.49 mmol/L and −6.29 ± 3.92 mmol/L), BIAsp 30 + BI group (−2.44 ± 1.52% points, −4.01 ± 2.50 mmol/L and −7.10 ± 3.96 mmol/L), BIAsp 30 + GI group (−2.33 ± 1.41% points, −4.34 ± 2.52 mmol/L and −7.97 ± 3.99 mmol/L) and BIAsp 30 + BI + TZD group (−1.21 ± 1.60% points, −3.50 ± 2.29 mmol/L and −5.97 ± 3.39 mmol/L). The proportion of patients who achieved an HbA_1c_ of less than 7% at three months was as follows: BIAsp 30-only (55%), BIAsp 30 + BI (49%), BIAsp 30 + GI (61%), and BIAsp 30 + BI + TZD (19%). Further, a small proportion of patients achieved target HbA_1c_ without reporting hypoglyceamic episodes (ranging from 47% to 64% across the groups). Based on the generalised linear model, duration of diabetes, baseline HbA_1c_, total daily dose of BIAsp 30 per bw, previous OAD treatment, and current treatment were found to have significant effects on the change in HbA_1c_ at three months from baseline (*P* < 0.01).

### Safety

The only serious ADR (classified under the category of unspecified events) in the “all exclude China” group was reported in the BIAsp 30-only group. Non-serious ADRs were reported in the BIAsp 30-only group (6 events), BIAsp + BI group (53 events) and BIAsp 30 + SU + BI group (28 events). The ADRs were classified as symptoms of hypersensitivity, acute painful neuropathy, refraction disorders, worsening of diabetic retinopathy, lipodystrophy, oedema and other unspecified events. In the “China” group, all ADR were non-serious in nature and reported in the BIAsp 30-only group (11 events), BIAsp 30 + BI group (9 events), BIAsp 30 + GI group (2 events) and none were reported in the BIAsp 30 + BI + TZD group. The ADRs were classified as symptoms of hypersensitivity, lipodystrophy or oedema.

**Figure 1:**
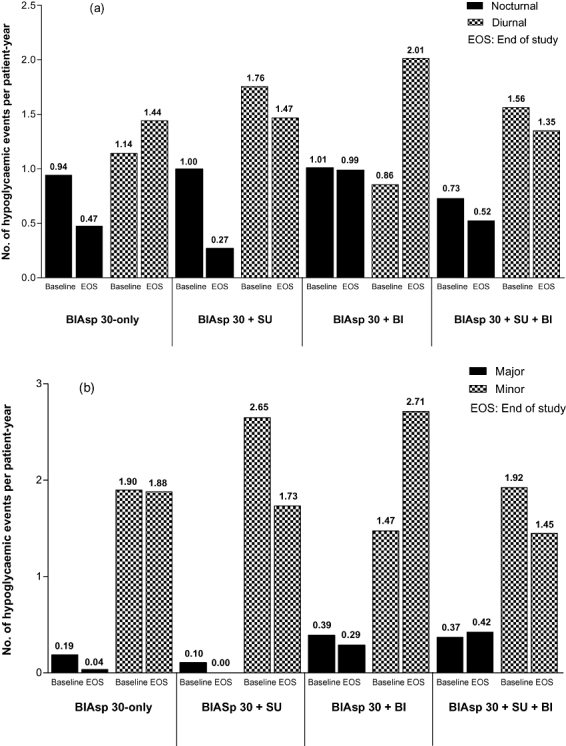
Hypoglycaemia at baseline and end of study stratified according to (a) time of day and (b) severity. End of study (EOS) includes data from three and six months—all exclude China.

In “all exclude China”, most of the hypoglycaemic episodes were reported at three months (1,303 episodes in the BIAsp-only group, 175 episodes in the BIAsp 30 + SU group, 880 episodes in the BIAsp 30 + BI group and 190 episodes in the BIAsp 30 + SU + BI group) and fewer episodes were reported at six months (963, 46, 637 and 239 episodes, respectively). When expressed as the number of episodes/patient year, the number of hypoglycaemic episodes/patient year at the end of study vs. baseline were as follows: BIAsp 30-only group (1.91 vs. 2.08), BIAsp 30 + BI group (3.00 vs. 1.86), and BIAsp 30 + SU group (1.73 vs. 2.75), BIAsp 30 + SU + BI group (1.87 vs. 2.29). Generally the most frequent hypoglycaemic episodes were diurnal and minor in nature (Figure 1). The rate of diurnal episodes increased from baseline in the BIAsp 30-only and BIAsp 30 + BI group. The rate of major episodes in the BIAsp 30 + SU + BI group increased marginally from baseline.

**Figure 2:**
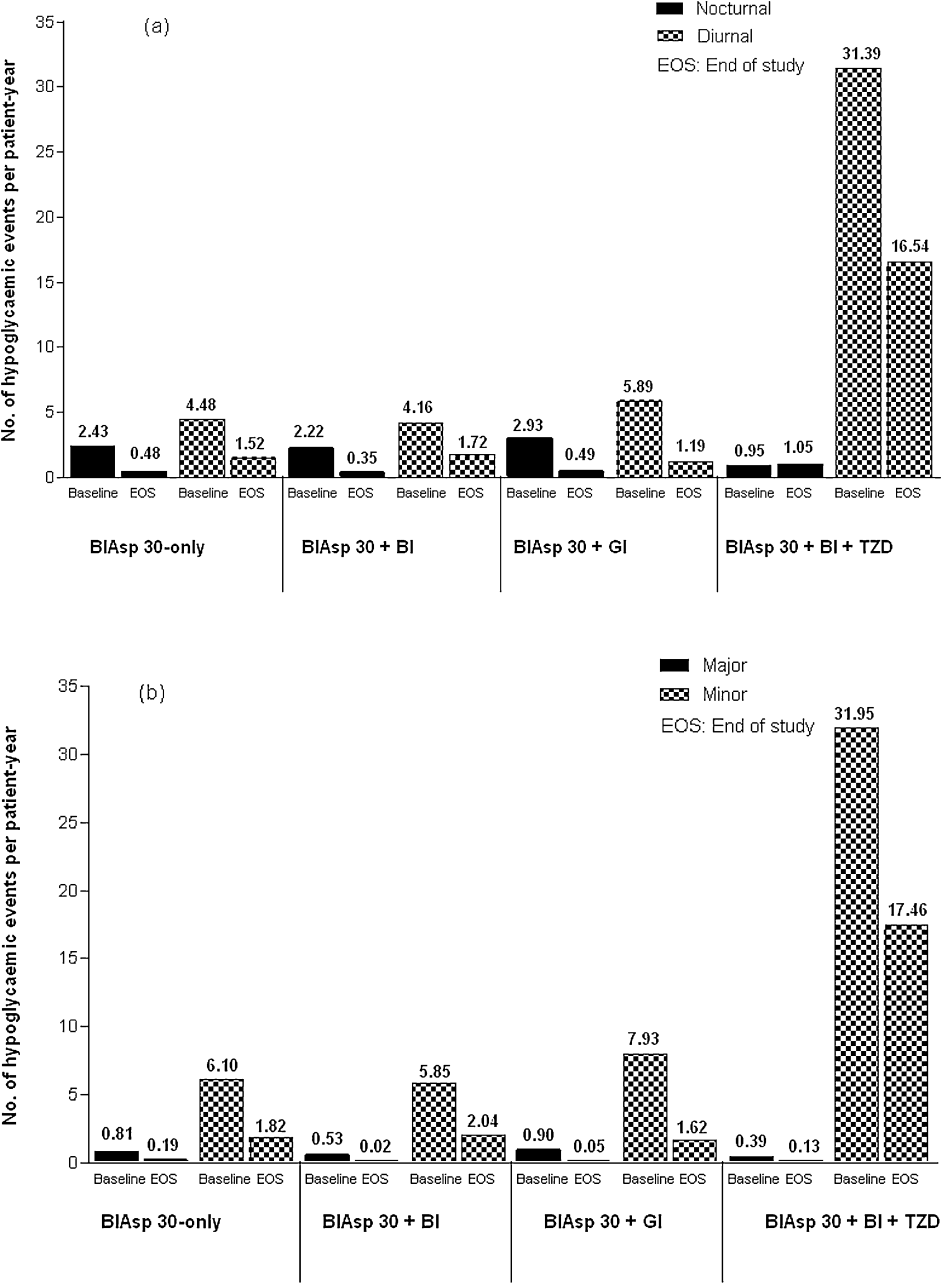
Hypoglycaemia at baseline and end of study stratfied according to (a) time of day and (b) severity—China.

In the “China” group, the number of hypoglycaemic episodes at three months were as follows: 1,237 episodes in the BIAsp-only group, 279 episodes in the BIAsp 30 + BI group, 69 episodes in the BIAsp 30 + GI group and 686 episodes in the BIAsp 30 + BI + TZD group. When expressed as the number of episodes/patient year, the number of hypoglycaemic episodes/patient year at the end of study vs. baseline were as follows: BIAsp 30-only group (2.01 vs. 6.91), BIAsp 30 + BI group (2.06 vs. 6.38), and BIAsp 30 + GI group (1.67 vs. 8.82), BIAsp 30 + BI + TZD group (17.59 vs. 32.33). Generally the most frequent hypoglycaemic episodes were diurnal and minor in nature (Figure 2). The rate of nocturnal episodes increased slightly from baseline in the BIAsp 30 + BI + TZD group.

## Discussion

Epidemiological analysis of data from the UKPDS study has shown that for every 1% point reduction in HbA_1c_, the risks of microvascular complications and myocardial infarction are reduced by 35% and 18%, respectively [3,19,20]. The mean HbA_1c_ in the insulin monotherapy and combination therapy groups in this study were all reduced by approximately 2% points. This observation suggests that the reductions in HbA_1c_ achieved by patients receiving BIAsp could be associated with decreased risks of diabetes-related complications but this will need to be formally tested.

The improvements in HbA_1c_ in this study were consistent with the observations of a 16-week randomised controlled trial of BIAsp 30 treatment in patients with type 2 diabetes [16]. In that study, the mean HbA_1c_ was reduced by 1.6% points in patients treated with insulin monotherapy and by 1.7% points in patients treated with insulin plus metformin.

The glucose-lowering effectiveness of individual therapies and combinations is dependent not only on the intrinsic characteristics of the intervention, but also on the baseline glycaemia, duration of diabetes, previous therapy and other factors [4]: many of these were not controlled in the present study and thus comparisons in effectiveness across the different intervention groups is not meaningful. However, it is noteworthy that diabetes control was improved in all the groups studied.

### Treatment Regimen

Although all the glucose parameters showed significant improvements at the end of the study, a substantial proportion of patients did not achieve the ADA recommended HbA_1c_ target of less than 7%. This was probably because the study was not conducted with a trial-to-target regimen and the insulin dosage was not optimised. The eventual dose requirements for patients with type 2 diabetes around the world is expected to be 50 to 100 U per day [21], which is much higher than the average doses for our patients.

## Limitations

This study was observational in nature and hence had its inherent limitations. Patients were not randomised to different therapies, nor was the dose of insulin or other anti-diabetic agents controlled. Thus, it is not possible to compare the responses across the groups in a meaningful manner. The method of data collection for hypoglycaemic episodes and ADRs was based on patient recollection, which could have resulted in under-reporting. Further, blood glucose measurements were not recorded during hypoglycaemic episodes that occurred prior to the start of the study. The study was conducted over a short period and was inadequate for capturing long-term trends and observations. Therefore, the results should be taken with some caution. However, the improvement in measure of diabetes control in this large number of patients enrolled in this study are concordant with findings from randomised controlled trials. Further, this study was carried out in a clinical setting, which would be a closer reflection of the “real-world” experience, as compared to highly selected patients clinical trials.

## Conclusions

The initiation of BIAsp 30 treatment in a clinical setting, either in as a monotherapy or in combination with OADs, was observed to improve glycaemic control in patients with type 2 diabetes who were poorly controlled on oral agents. This improvement was accompanied with a low occurrence of major and nocturnal hypoglycaemic episodes.

## References

[1] Shestakova M, Lebedev N, Kedijang T, Tuna S, Hassan A, Shinde A (2007). Improved glycemic control (hba_1c_ < 7%) and reduced hypoglycemic episodes with biphasic insulin aspart 30 treatment in type 2 diabetes mellitus patients poorly controlled on OADs: Results from the PRESENT study. Diabetes.

[2] Turner RC, Cull CA, Frighi V, Holman RR (1999). Glycemic control with diet, sulfonylurea, metformin, or insulin in patients with type 2 diabetes mellitus: Progressive requirement for multiple therapies (UKPDS 49). UK Prospective Diabetes Study (UKPDS) Group. JAMA.

[3] UK Prospective Diabetes Study (UKPDS) Group (1998). Intensive blood-glucose control with sulphonylureas or insulin compared with conventional treatment and risk of complications in patients with type 2 diabetes (UKPDS 33). Lancet.

[4] Nathan DM, Buse JB, Davidson MB, Heine RJ, Holman RR, Sherwin R (2006). Management of hyperglycemia in type 2 diabetes: A consensus algorithm for the initiation and adjustment of therapy: A consensus statement from the American Diabetes Association and the European Association for the Study of Diabetes. Diabetes Care.

[5] Nathan DM, Roussell A, Godine JE (1988). Glyburide or insulin for metabolic control in non-insulin-dependent diabetes mellitus. A randomized, double-blind study. Ann Intern Med.

[6] Wright A, Burden AC, Paisey RB, Cull CA, Holman RR (2002). Sulfonylurea inadequacy: Efficacy of addition of insulin over 6 years in patients with type 2 diabetes in the U.K. Prospective Diabetes Study (UKPDS 57). Diabetes Care.

[7] Goudswaard AN, Furlong NJ, Rutten GE, Stolk RP, Valk GD (2004). Insulin monotherapy versus combinations of insulin with oral hypoglycaemic agents in patients with type 2 diabetes mellitus. Cochrane Database Syst Rev.

[8] Kilo C, Mezitis N, Jain R, Mersey J, McGill J, Raskin P (2003). Starting patients with type 2 diabetes on insulin therapy using once-daily injections of biphasic insulin aspart 70/30, biphasic human insulin 70/30, or NPH insulin in combination with metformin. J Diabetes Complications.

[9] Leiter LA, Ceriello A, Davidson JA, Hanefeld M, Monnier L, Owens DR (2005). Postprandial glucose regulation: New data and new implications. Clin Ther.

[10] Christiansen JS, Vaz JA, Metelko Z, Bogoev M, Dedov I (2003). Twice daily biphasic insulin aspart improves postprandial glycaemic control more effectively than twice daily NPH insulin, with low risk of hypoglycaemia, in patients with type 2 diabetes. Diabetes Obes Metab.

[11] Raskin P, Allen E, Hollander P, Lewin A, Gabbay RA, Hu P (2005). Initiating insulin therapy in type 2 diabetes: A comparison of biphasic and basal insulin analogs. Diabetes Care.

[12] Jacobsen LV, Sogaard B, Riis A (2000). Pharmacokinetics and pharmacodynamics of a premixed formulation of soluble and protamine-retarded insulin aspart. Eur J Clin Pharmacol.

[13] Garber AJ (2006). Premixed insulin analogues for the treatment of diabetes mellitus. Drugs.

[14] Boehm BO, Home PD, Behrend C, Kamp NM, Lindholm A (2002). Premixed insulin aspart 30 vs. premixed human insulin 30/70 twice daily: A randomized trial in Type 1 and Type 2 diabetic patients. Diabet Med.

[15] Boehm BO, Vaz JA, Brondsted L, Home PD (2004). Long-term efficacy and safety of biphasic insulin aspart in patients with type 2 diabetes. Eur J Intern Med.

[16] Kvapil M, Swatko A, Hilberg C, Shestakova M (2006). Biphasic insulin aspart 30 plus metformin: An effective combination in type 2 diabetes. Diabetes Obes Metab.

[17] Raz I, Mouritzen U, Vaz J, Hershkovitz T, Wainstein J, Harman-Boehm I (2003). Addition of biphasic insulin aspart 30 to rosiglitazone in type 2 diabetes mellitus that is poorly controlled with glibenclamide monotherapy. Clin Ther.

[18] Khutsoane D, Sharma SK, Almustafa M, Jang HC, Azar ST, Danciulescu R (2008). Biphasic insulin aspart 30 treatment improves glycaemic control in patients with type 2 diabetes in a clinical practice setting: Experience from the PRESENT study. Diabetes Obes Metab.

[19] American Diabetes Association (2003). Implications of the United kingdom prospective diabetes study. Diabetes Care.

[20] Bretzel RG, Voigt K, Schatz H (1998). The United Kingdom Prospective Diabetes Study (UKPDS) implications for the pharmacotherapy of type 2 diabetes mellitus. Exp Clin Endocrinol Diabetes.

[21] International Diabetes Federation Clinical Guidelines Task Force (2006). Global guideline for type 2 diabetes: Recommendations for standard, comprehensive and minimal care. Diabet Med.

